# Limited evidence and ethical considerations in rTMS trials for adolescents with obsessive–compulsive disorder

**DOI:** 10.1192/bjb.2025.10138

**Published:** 2025-12

**Authors:** Raphael J. Leo, Brandon L. Mariotti

**Affiliations:** Department of Psychiatry, University at Buffalo, Jacobs School of Medicine and Biomedical Sciences, NY, USA

**Keywords:** Obsessive–compulsive disorder, adolescence, rTMS

## Abstract

Repetitive transcranial magnetic stimulation (rTMS) has gained regulatory approval as an adjunctive treatment for obsessive–compulsive disorder (OCD) in adults. However, its application in adolescents remains largely untested. This editorial examines the limited evidence available, focusing on choice of target, stimulation depth and methodological variation. Ethical challenges surrounding the use of rTMS in vulnerable populations, including informed consent and the unknown long-term effects on neurodevelopment, are also discussed. Although rTMS holds promise for treatment-resistant adolescent OCD, a cautious and ethically rigorous approach is essential before wider clinical adoption can be considered.

Repetitive transcranial magnetic stimulation (rTMS) is a promising non-invasive neuromodulation technique for treatment-resistant psychiatric conditions, including obsessive–compulsive disorder (OCD). rTMS utilises repetitive electromagnetic pulses to modulate neural activity in targeted brain regions. A variant, deep TMS (dTMS), employs H-coils designed to penetrate deeper cortical and subcortical structures. Both approaches are generally well tolerated and do not require anaesthesia, distinguishing them from electroconvulsive therapy, which induces generalised seizures and carries different cognitive side-effects. While dTMS has received regulatory approval (U.S. Food and Drug Authority in the USA, Conformité Européenne mark in Europe) as an adjunctive therapy for adult OCD, its use in adolescents remains investigational.

The prevalence of OCD among paediatric populations is estimated to be 1–2%.^1^ Often emerging during childhood or adolescence, OCD is associated with significant functional impairment. Although cognitive–behavioural therapy and selective serotonin reuptake inhibitors remain first-line treatments, a notable proportion of young patients experience only partial symptom remission and persistent impairments.^1^ In this context, rTMS has attracted growing interest as a potential therapeutic option. However, the limited empirical evidence and significant ethical concerns both underscore the need for caution in extending rTMS to this vulnerable population.

In adults, low-frequency rTMS targeting the supplementary motor area (SMA) has shown promise in alleviating symptoms through modulation of cortico-striato-thalamo-cortical (CSTC) networks.^2^ The SMA, a key node in motor control and habit formation, is implicated in the perpetuation of compulsive behaviours. This target is also common in rTMS studies of Tourette’s disorder, a condition that frequently co-occurs with OCD and shares overlapping circuitry involving motor inhibition.^3^ However, a small case series of adults with Tourette’s and comorbid OCD treated with SMA-targeted rTMS did not address improvements in OCD-specific symptoms, highlighting a gap in the literature concerning comorbid presentations.^4^

Empirical evidence supporting rTMS in adolescent OCD remains limited (see Table 1). Among the few publications addressing refractory OCD among adolescents, the dorsolateral prefrontal cortex (DLPFC) has been a frequent target for treatment. The DLPFC is easily accessible and has been well studied in adolescent populations for mood and anxiety disorders. It plays a critical role in cognitive–emotional regulation and connects to deeper nodes within the CSTC circuit, such as the anterior cingulate cortex (ACC), striatum and thalamus. Consequently, DLPFC stimulation may indirectly modulate activity in SMA-related pathways via prefrontal–striatal connections.

**Table 1:** Summary of reports addressing rTMS in adolescent OCD

Author(s)	*N*	Age/gender	rTMS modality	Target	Outcome measure(s)	Outcome summary
Das et al^7^	1	17 y/F	Deep rTMS, 20 HF sessions (50% MT, 2000 pulses)	mPFC, ACC	Y-BOCS	60% reduction in symptoms after 20 sessions
Hegde et al^6^	1	16 y/M	Conventional rTMS, 18 LF sessions (100% MT, 1200 pulses)	DLPFC, pre-SMA	Y-BOCS, CGI-S	No significant improvement
Pedapati et al^5^	18	1 M/7 F (sham); 5 M/5 F (treatment)	Conventional TMS, 1 LF single-pulse session (110% MT, 1800 pulses)	DLPFC	Y-BOCS	Minimal change in OCD symptoms
Sharma et al^8^	1	17 y/F	Deep rTMS + tDCS, 30 HF sessions (50% MT, 2000 pulses)	mPFC, ACC	Y-BOCS, CGI-S	60% reduction in symptoms after 30 sessions

rTMS, repetitive transcranial magnetic stimulation; OCD, obsessive–compulsive disorder; F, female; M, male; HF, high-frequency; MT, motor threshold; mPFC, medial prefrontal cortex; ACC, anterior cingulate cortex; LF, low-frequency; DLPFC, dorsolateral prefrontal cortex; pre-SMA, presupplementary motor area; tDCS, transcranial direct current stimulation; Y-BOCS, Yale–Brown Obsessive–Compulsive Scale; CGI-S, Clinical Global Impression – Severity Scale.

In the only published study of rTMS involving multiple adolescent patients with OCD, functional magnetic resonance imaging was utilised to investigate activity changes in a variety of neural circuits presumed to be implicated in OCD during active or sham rTMS targeted to the right DLPFC.^5^ Although this study provided an initial framework for TMS interrogation of the CSTC circuitry and paediatric OCD, only two of ten patients in the active-treatment condition demonstrated an appreciable change in OCD symptoms. However, it is noteworthy that this study utilised only a single session of rTMS. Typically reserved for research or diagnostic purposes, single-session rTMS does not align with standard protocols for clinical treatment of OCD, which customarily entails repeated sessions over several weeks. Additionally, the limited depth of penetration inherent to the figure-of-eight coils employed in this study may have accounted for the lack of appreciable clinical benefit. Although the study demonstrated that rTMS can acutely modulate CTSC-related activity, its shallow stimulation range may have failed to engage deeper nodes of the OCD circuit, i.e. the SMA or ACC. This is consistent with findings from a case report in which a similar coil and superficial pre-SMA targeting did not result in symptom relief.^6^

While still based on limited evidence, recent case reports using dTMS have shown therapeutic potential in adolescents.^7,8^ Rapid improvements in obsessions, compulsions and overall illness severity were achieved in a 17-year-old female following stimulation of the medial prefrontal and ACC.^7^ In another case, high-definition transcranial direct current stimulation was coupled with DLPFC-targeted dTMS, with the former priming the latter in each session, to induce a reduction in OCD symptoms in a 17-year-old female described as ‘ultra-treatment-resistant’.^8^ These observations suggest that the efficacy of rTMS in paediatric OCD depends not only on the choice of target but also on the depth and breadth of stimulation afforded by coil design. Aside from mild, transient headaches following a minority of sessions, dTMS and rTMS were well tolerated, with no major adverse effects.

Despite mildly promising early efforts, the field lacks well-powered, randomised controlled trials (RCTs) that rigorously assess short- or long-term efficacy. Reports vary substantially in TMS modality and stimulation protocols, including targeted brain region and the frequency, intensity and duration of treatment. Methodologic standardisation will be necessary to determine potential benefits in paediatric populations.

Beyond empirical limitations, the ethical implications of rTMS research in adolescence merit discussion.^9^ First is the challenge of informed consent and assent. Due to their developmental stage, adolescents may lack the full capacity to comprehend the experimental nature of rTMS, especially in the context of treatment resistance and heightened parental hope. The potential for therapeutic misconception is a significant concern, particularly in cases refractory to first-line treatments.

While rTMS is generally well tolerated in adults, its safety in the developing brain is not fully established. Given the ongoing maturation of cortical circuits, adolescents may be uniquely vulnerable to unintended neurodevelopmental effects. Although adverse events in paediatric rTMS studies have typically been mild (i.e. headache, scalp discomfort), there are insufficient data to assess long-term neurocognitive or developmental consequences.

Ethical questions also arise regarding the use of sham control conditions and trials involving symptomatic adolescents with limited treatment options. While sham-controlled RCTs are essential for scientific rigour, prolonged placebo exposure in a vulnerable population raises concerns about non-maleficence. The risk–benefit ratio of such trials must be scrutinised for scientific integrity and strong ethical safeguards. Trials should include developmentally sensitive protocols, thorough safety monitoring, long-term follow-up, independent ethical oversight and, perhaps most importantly, transparency in communication with participants and families about the experimental nature of rTMS. If effective, protocols should include provisions for post-trial access to active treatment, e.g. open-label extensions for sham participants, to mitigate ethical concerns and enhance participant trust.

In conclusion, while rTMS holds promise for adult OCD, there is a crucial gap in our understanding of TMS as a treatment modality in paediatric populations with OCD. A more robust and ethically grounded research agenda is essential before rTMS can be considered a viable therapeutic tool for this vulnerable population. Despite growing interest in the application of rTMS for psychiatric conditions, the evidence supporting its use, particularly for dTMS, in paediatric populations for OCD remains limited.

## About the authors

**Raphael J. Leo**, MA, MD, Associate Professor, Department of Psychiatry, University at Buffalo, Jacobs School of Medicine and Biomedical Sciences, Buffalo, New York, USA. **Brandon L. Mariotti**, MD, MA, Assistant Clinical Instructor Combined Adult/Child Psychiatry Residency Program, Department of Psychiatry, University at Buffalo, Jacobs School of Medicine and Biomedical Sciences, Buffalo, New York, USA
